# Competing interplay between systemic and periodontal inflammation: obesity overrides the impact of oral periphery

**DOI:** 10.1007/s00784-020-03514-y

**Published:** 2020-08-22

**Authors:** Peter Meisel, Christiane Pink, Vinay Pitchika, Matthias Nauck, Henry Völzke, Thomas Kocher

**Affiliations:** 1 Dental Clinics, Department of Periodontology, School of Dentistry, University Medicine Greifswald, Fleischmann-Strasse 42, D-17475 Greifswald, Germany; 2 Institute of Clinical Chemistry and Laboratory Diagnostics, Greifswald, Germany; 3 DZHK (German Centre for Cardiovascular Research), Partner Site Greifswald, University Medicine, Greifswald, Germany; 4 Institute for Community Medicine, University Medicine Greifswald, Greifswald, Germany

**Keywords:** Obesity, Periodontitis, Inflammation, C-reactive protein

## Abstract

**Objectives:**

We aimed at investigating whether the interaction between the local inflammation, periodontitis, and obesity is independently associated with systemic inflammation.

**Methods:**

From the population-based Study of Health in Pomerania, 3366 participants, without (2366) and with (1000) obesity, were studied for the association of periodontitis, measured as probing depth (PD) and plaque together with body mass index (BMI) on C-reactive protein (CRP). Quantile regression was used to evaluate the association between periodontal, anthropometric, and inflammatory variables (outcomes).

**Results:**

The overall prevalence of obesity in this adult population was 31.4% in men and 28.1% in women. Both PD and plaque were positively associated with CRP, revealing an increasing impact across the CRP concentration distribution. Adjusting the regression of CRP or fibrinogen on PD for waist circumference attenuated but did not abolish the PD coefficients. Dental plaque was similarly associated with these interrelations. Association between PD and a dental plaque was different among participants with low-, medium-, or high-risk CRP concentrations.

**Conclusion:**

Local and systemic sources of inflammation contribute to blood levels of inflammatory markers. The respective contributions depend on the relative rate in each of the inflammation-inducing risks and are dominated by adiposity.

**Clinical relevance:**

Keeping systemic inflammation low in order to prevent age-related disease sequelae.

**Electronic supplementary material:**

The online version of this article (10.1007/s00784-020-03514-y) contains supplementary material, which is available to authorized users.

## Introduction

Periodontitis is an inflammatory chronic disease of the gum leading to attachment loss and bone destruction. Periodontal disease may eventually result in tooth loss and is associated with systemic inflammatory diseases. Various studies of the recent decade offer facts and correlations between obesity and periodontitis, which appear controversial and often difficult to explain at first glance. Nevertheless, it is obvious that obesity is associated with the rate and severity of periodontitis and, on the other hand, periodontitis may affect the risk of obesity [1].

The increasing prevalence of overweight and obesity worldwide presents a major health concern as it interferes with health conditions across the life course. Obese individuals have an increased risk of multiple chronic diseases such as type 2 diabetes, cardiovascular diseases, and other inflammatory sequelae, among them periodontitis [2, 3]. Furthermore, various studies have associated periodontitis with diabetes and cardiovascular diseases, too [4]. Besides these relationships, elevated systemic concentrations of inflammatory markers are associated with obesity as well. Obesity is characterized by systemic inflammation and this is reflected in higher levels of CRP, which is closely related to total body fat [5, 6]. Elevated levels of systemic inflammation may increase the likelihood of periodontal sequelae by decreasing thresholds for invading inflammatory cells. Conversely, elevated blood levels of CRP or IL-6 are commonly observed in periodontitis, indicating systemic reactions to the local infection and inflammation within the oral cavity [7, 8].

Today, predominant opinion connects the local inflammation of periodontitis with chronic systemic inflammatory diseases via mediating pathways characterized by various indicators of inflammation such as fibrinogen, C-reactive protein (CRP), and interleukin IL-6.

A mutual relationship might exist among obesity, CRP, and periodontitis [8, 9]. Visceral fat in obesity plays a major role in the development of metabolic disturbances associated with systemic diseases, eventually interacting with the local inflammation in periodontal diseases.

Thus, as obesity and elevated CRP levels are associated with multiple chronic diseases [10], it is believed that inflammatory markers play a mediating role in their etiology. An enormously complex network of intertwined and competing mechanisms of local and systemic inflammatory diseases is the consequence, complicating the search for a causal direction of disease interplay. Moreover, the aging process involves a low-grade chronic systemic inflammation. Obesity superimposed on aging drastically increases chronic low-grade inflammation (inflammaging), which is an important link between obesity- and age-associated diseases, e.g., insulin resistance being also associated with periodontitis [11, 12].

Complicating the complex interactions, cytokine levels are differently affected by obesity markers such as body mass index (BMI), waist circumference, or fat mass according to sex [13, 14].

While an association between a reduced number of teeth and increased CRP levels was found in some studies, this association varied across sexes and BMI categories [7, 15]. However, it still remains unclear how periodontitis and obesity are associated with their interdependent contribution to systemic inflammation. It is still controversial whether their mutual contribution to systemic inflammation maybe just coincidentally or causally connected.

Thus, it is the objective of this study to assess the quandaries in the relationship between the systemic and local sources of CRP and fibrinogen from adiposity and from periodontitis, respectively.

## Material and methods

### Study design and sample

The second Study of Health in Pomerania (SHIP-Trend) is a population-based project conducted in the Northeast of Germany. Baseline examinations of this cross-sectional study cohort were conducted between 2008 and 2012 [16]. Out of 8016 eligible adults enrolled in the SHIP-Trend, 4420 agreed to participate and underwent comprehensive examinations. For this study, a number of those participants were excluded in our final analyses based on exclusion criteria such as complete edentulism, missing clinical data or refused answers in questionnaires or otherwise missing data, leaving 3366 participants (1000 and 2366 participants with and without obesity, respectively). All participants gave their written informed consent and the study was approved by the local ethics committee.

### Periodontal examinations and independent variables

The periodontal assessment included probing depth (PD), clinical attachment level (CAL), dental plaque, bleeding on probing, and the number of teeth. For the number of teeth, all fully erupted teeth were assessed excluding third molars (maximum 28). The periodontal examination was carried out on either the left- or right-side quadrants and the examination side was changed from participant to participant. A maximum of 14 teeth per participant was examined. Clinical attachment level (CAL) and probing depth (PD) were assessed with a periodontal probe (PCP 15, HuFriedy, Chicago IL) at mesiobuccal, distobuccal, midbuccal, and midlingual aspect on each selected tooth and recorded as whole millimeters. We used mean PD in millimeters to characterize the acute state of periodontitis and body mass index (BMI, kg/m^2^) and sex-specific waist circumference (WC) for adiposity. Plaque is a rather rough indicator of dental hygiene and the presence of supragingival biofilm. It was registered as a percentage of affected sites detected, categorized into tertiles. Indicators of sociodemographic variables were obtained from the health-related interviews or the personal questionnaire (education, smoking). The level of education was categorized according to the final school grade (< 10 years, 10 years, > 10 years). Smoking was categorized into never, former, and current smokers. Anthropometric measurements were taken under standardized conditions using balance and height measuring devices (SOEHNLE, Murrhardt, Germany). Body weight was measured to the nearest 0.10 kg on a decimal scale, height to the nearest 1 cm, and waist and hip circumferences to the nearest 0.5 cm. Waist girth was measured at the midpoint between the lower ribs and the iliac crest. Hip circumference was measured horizontally at the level of the largest lateral extension of the hips or over the buttocks. A non-fasting blood sample was drawn from the antecubital vein in the supine position and immediately analyzed or stored at − 80 °C. Fibrinogen and glycated hemoglobin (HbA1c) were measured with standard laboratory methods. High sensitivity CRP was determined in serum by particle-enhanced immuno-nephelometry (hsCRP kit, Dade Behring Inc.) with a test sensitivity of 0.2 mg/L. Handgrip strength was used as a proxy for the general health state of the participants, which is strongly associated with obesity [17]. Handgrip strength was measured by handheld Smedley-type dynamometer used for diagnostic purposes (Scandidact, Denmark); it was indicated in kilograms. We measured grip strength left and right handed and used the maximum strength of either hand as an independent variable.

### Statistical analyses

Mann-Whitney tests or contingence tables were used to assess obesity-specific differences in continuous and categorical variables, respectively. We used multivariate quantile regression analyses to evaluate the association between adiposity-related, periodontal, and inflammatory variables (outcomes) and independent covariates [18]. Quantile regression was used to model the effects of covariates on the conditional quantiles of an outcome variable. This approach is a robust method that makes no distributional assumption about the error term and is insensitive to outliers. Conventionally, concentrations above 10 mg/L were excluded to avoid bias from acute inflammation. Higher CRP levels were included as obese women were concerned [19]. We used Stata/SE 14.2 software (Stata Corp LP, College Station, TX, USA).

## Results

### Obesity at baseline

Introducing the underlying problem, we demonstrate the considerable differences in all assessed parameters between participants with obesity and those without (Table 1). The table presents the baseline characteristics of the study population separated at the BMI cutoff of 30 kg/m^2^. The overall prevalence of obesity under the exclusion criteria of this study was 31.4% in men (*N* = 518) and 28.1% (*N* = 482) in women. The extent of the plaque was nearly double in participants with obesity as compared with their leaner counterparts. The median of CRP in this population was different in as much as between 1.0 and 2.3 mg/L in non-obese and obese participants; the median of the number of teeth was 25 and 22 teeth, respectively. The comparison between CRP and PD means and medians reveals that in both CRP and PD approximately 65–70% of the participants were at much lower levels of these markers than the average. With BMI, this figure was merely 55%, higher in women and less in men. Though similarly affected by the obesity burden, there is no explanation for a common mechanism behind the shift in these distributions for CRP and teeth as the mean age difference was merely odd 6 years. Higher age may be associated with less education, obesity, and fewer smokers. PD characterizes the actual periodontal inflammation differing considerably between obese and non-obese participants. The colonization with dental plaque was also significantly different between non-obese and obese participants. This also held true for systemic measures of inflammation such as CRP and fibrinogen as well as periodontal parameters CAL and PD. Mean handgrip strength was not different between both groups. It was included as a proxy of general health.

**Table 1:** Baseline characteristics of the participants with complete data set (*N* = 3332)

	BMI < 30 kg/m^2^ (*N* = 2366)	BMI ≥ 30 kg/m^2^ (*N* = 1000)
Age, years	48.7 ± 15.0	54.6 ± 13.2
Female participants (%)	1,232 (52.1)	482 (48.2)
HbA1C, %	5.2 ± 0.7	5.6 ± 0.9
Participants with HbA1c **≥** 6.5%	76 (3.2)	112 (11.2)
Mean CRP, mg/L	1.9 ± 3.7	3.8 ± 6.8
Median CRP, mg/L (IQR)	1.0 (0.5–2.1)	2.3 (1.2–4.5)
Subjects with CRP > 3 mg/L	378 (16.0)	390 (39.0)
Fibrinogen, g/L	2.9 ± 0.7	3.3 ± 0.8
Body mass index (BMI, kg/m^2^)	25.4 ± 2.8	34.0 ± 3.9
Waist-to-hip ratio (WHR)—male	0.92 ± 0.06	1.00 ± 0.06
- Female	0.81 ± 0.06	0.86 ± 0.06
Waist circumference (WC), cm—male	91.0 ± 8.3	109.6 ± 9.7
- Female	78.6 ± 8.2	100.3 ± 10.1
Handgrip strength, kg—male	47.4 ± 8.5	47.6 ± 8.9
- Female	28.8 ± 5.7	28.6 ± 6.8
Mean PD, mm	2.5 ± 0.6	2.8 ± 0.8
Median PD, mm (IQR)	2.4 (2.1–2.7)	2.6 (2.3–3.0)
Extent PD ≥ 4 mm, median (IQR)	5.4 (0–17.5)	11.1 (2.1–28.1)
Mean CAL, mm	2.3 ± 1.6	2.9 ± 1.8
Plaque, median % of sites (IQR)	12.5 (0–33.3)	22.9 (5.0–54.2)
No. of teeth, mean	22.3 ± 6.5	19.7 ± 7.5
No of teeth, median (IQR)	25 (20–27)	22 (15–26)
Education, less than 10th grade (%)	371 (15.7)	255 (25.5)
Smoker, current (%)	602 (25.4)	188 (18.8)

Data are presented as number (percent), mean ± SD, and/or median (IQR, 25–75% quantile)

### Competing CRP origins

As mentioned, differences between mean and median concentrations of CRP were observed. For the subsequent analyses, we focused on periodontal measures, especially on PD as it represents acute inflammation better than CAL, which is accumulating with age. A comparison of case distributions within the quartiles of PD as well as of CRP revealed the tendency of increasing dispersion with higher figures in both (not shown). To focus on the typical people at the median, comprising the interquartile range, we performed a quartile regression on CRP concentrations using relevant independent variables, especially PD, as presented in Table 2.

**Table 2:** Quartile regression on 25th, 50th, and 75th percentile of C-reactive protein (CRP, mg/L) with PD, *N* = 3366; *ß* coefficients with 95% confidence intervals

Independent variables	1st quartile	Median	3rd quartile
Crude			
Age, 10-year increments	0.05 (0.04; 0.07)	0.08 (0.03; 0.13)	0.08 (− 0.05; 0.22)
Female sex (ref. male)	0.13 (0.08; 0.18)	0.38 (0.26; 0.51)	1.02 (0.62; 1.37)
Mean PD, mm	0.15 (0.10; 0.20)	0.32 (0.20; 0.44)	0.58 (0.35; 0.82)
Model 1			
Age, 10-year increments	− 0.03 (− 0.04; − 0.01)	− 0.10 (− 0.12; − 0.07)	− 0.21 (− 0.29; − 0.14)
Female sex (ref. male)	0.52 (0.46; 0.59)	1.00 (0.92; 1.08)	1.95 (1.75; 2.14)
Mean PD, mm	0.06 (− 0.00; 0.12)	0.18 (0.11; 0.25)	0.46 (0.30; 0.63)
Waist circumference, cm	0.03 (0.03; 0.03)	0.05 (0.05; 0.06)	0.09 (0.08; 0.11)
Model 2*			
Age, 10 years increments	− 0.04 (− 0.06; − 0.02)	− 0.11 (− 0.13; − 0.09)	− 0.28 (− 0.38; − 0.17)
Female sex (ref. male)	0.19 (0.06; 0.31)	0.41 (0.27; 0.55)	0.61 (0.12; 1.11)
Mean PD, mm	0.06 (0.02; 0.10)	0.13 (0.07; 0.20)	0.32 (0.15; 0.49)
Waist circumference, cm	0.02 (0.01; 0.02)	0.02 (0.01; 0.03)	0.04 (0.02; 0.06)
BMI, kg/m^2^	0.04 (0.02; 0.06)	0.10 (0.08; 0.12)	0.16 (0.11; 0.21)
HbA1c, %	0.08 (0.02; 0.13)	0.14 (0.06; 0.22)	0.27 (0.12; 0.41)
Handgrip strength, 5-kg steps	− 0.06 (− 0.08; − 0.03)	− 0.09 (− 0.11; − 0.06)	− 0.21 (− 0.32; − 0.11)

*Additionally adjusted for smoking in 3 categories, education in 3 categories

Coefficients of PD increased with increasing CRP levels across the CRP quartiles with age and female sex, both positively correlated to CRP. Adjusting for WC in the regression attenuated the PD coefficients by more than 50% but leaving them significantly associated with CRP (model 1). Further adjustment for important confounders such as BMI and HbA1c were of minor effect (model 2).

**Fig. 1:**
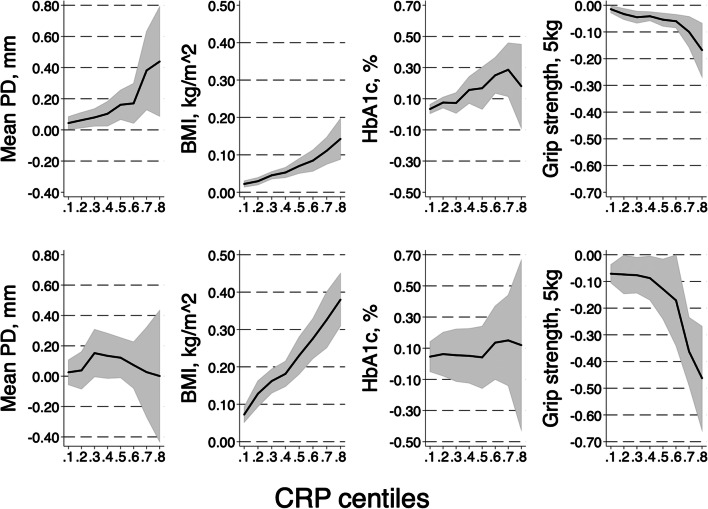
Regression on CRP quantiles according to models in Table 2. The ordinates indicate the regression coefficients relevant to each of the increasing CRP quantiles. Shaded areas are 95% confidence ranges. Results of regression in participants with BMI < 30 kg/m^2^ (upper row) and in obese participants (lower row)

Quantile regression was performed across the entire conditional distribution of CRP to illustrate the different impact of obesity (BMI ≥ 30 kg/m^2^) on the covariates. Graphical results are shown in Fig. 1. Clearly, the association of PD and waist circumference with CRP increases along with the CRP concentration range, most pronounced at levels above the CRP median (quantile 0.5 in Fig. 1). Under obese conditions, waist circumference was more important relative to PD whose effect was attenuated in comparison with non-obesity. In obesity, the impact of muscle strength, a proxy to characterize general health, exhibited a stronger effect and showed a negative association with CRP levels. The coefficients given at the ordinates are interpreted as the increase in CRP at each of its quantiles by a one-unit increase of the factor indicated at the ordinates (or decrease as in case of muscle strength). These coefficients are higher when the independent variables, obesity, PD, and sex are regressed on increasing CRP levels.

Both obesity and PD were associated with systemic CRP and fibrinogen concentrations. Thus, there were systemic contributions from obesity and local contributions from periodontitis to CRP blood levels indicating systemic inflammation. For comparison, a quartile regression was performed with fibrinogen as the dependent variable. Inclusion of WC also attenuated the PD coefficients for fibrinogen; however, they remained nearly equal across the quartiles, probably due to the nearly normal distribution of fibrinogen values. When stratified according to WHO criteria, high-risk WC abolished the PD contribution to fibrinogen at its mean completely (Table 3). Also here, the regression was adjusted for BMI.

**Table 3:** Median regression of fibrinogen (g/L) with PD as the independent variable; stratified by WHO WC risk categories (high-risk WC > 88 cm and > 102 cm in females and males), respectively), *ß* coefficients with 95% confidence intervals

Independent variables	Low WC risk *N* = 2238	*p*	High WC risk *N* = 1128	*p*
Age, 10-year increments	0.05 (0.02; 0.08)	< 0.001	0.03 (− 0.02; 0.08)	0.25
Female sex (ref. male)	0.28 (0.17; 0.38)	< 0.001	0.20 (0.03; 0.37)	0.022
Mean PD, mm	0.08 (0.03; 0.13)	0.004	0.04 (− 0.03; 0.11)	0.30
Number of teeth	− 0.01 (− 0.01; 0.00)	0.11	− 0.00 (− 0.01; 0.00)	0.34
BMI, kg/m^2^	0.03 (0.02; 0.04)	< 0.001	0.04 (0.03; 0.05)	< 0.001
HbA1c, %	0.28 (0.22; 0.33)	< 0.001	0.07 (0.02; 0.13)	0.008

Median regression is also adjusted for grip strength, smoking, education

In Table 4, an analogous regression on CRP is shown with the extent of dental plaque instead of PD. Though dental plaque is a rather crude measure of biofilm extent due to the superficial assessment, it was also used as an independent oral variable. The results correspond to PD figures related to CRP in Table 2, i.e., controlling for waist circumference (or also for BMI) attenuated the plaque coefficients (not shown). Moreover, stratifying the analyses according to WC risk categories revealed increased coefficients for plaque and also for BMI on CRP (Table 4). Similarly, as shown in Fig. 1, in participants with high WC, the negative association of muscle strength with CRP was augmented as compared with participants with normal WC.

**Table 4:** Median regression on 50th percentile of CRP as dependent variable depending on plaque tertiles, stratified by WC risk categories (high-risk WC > 88 cm and > 102 cm in females and males, respectively), *ß* coefficients with 95% confidence intervals

Independent variables*	Low WC risk *N* = 2238	*p*	High WC risk *N* = 1128	*p*
Age, 10-year increments	− 0.04 (− 0.08; 0.01)	0.15	− 0.15 (− 0.33; 0.02)	0.085
Female sex (ref. male)	0.16 (− 0.02; 0.34)	0.075	0.14 (− 0.41; 0.70)	0.61
Plaque, 1st tertile	0	Ref.	0	Ref.
Plaque, 2nd tertile	− 0.05 (− 0.16; 0.07)	0.45	0.14 (− 0.29; 0.56)	0.53
Plaque, 3rd tertile	0.15 (0.02; 0.29)	0.027	0.35 (− 0.08; 0.77)	0.11
BMI, kg/m^2^	0.07 (0.05; 0.09)	< 0.001	0.22 (0.19; 0.26)	< 0.001
HbA1c, %	0.17 (0.08; 0.26)	< 0.001	0.03 (− 0.14; 0.21)	0.71
Handgrip strength, 5=kg steps	− 0.05 (− 0.09; − 0.01)	0.012	− 0.15 (− 0.26; − 0.03)	0.014

*Also adjusted for the number of teeth, smoking, education

BMI (or obesity for that matter) was associated with higher CRP mainly in females and to a lesser extent also in males (not shown). The PD effect on CRP was more pronounced in men but in women, the anthropomorphic effects predominated. This distinction was also observed with a plaque as an independent variable.

Finally, the impact of obesity (in terms of WC) under different CRP risk levels was estimated for the association between dental plaque and PD, that is, between the extent of supragingival biofilm and the periodontal measure. The corresponding regression analysis is given in Table 5. Even when adjusted for WC, increasing CRP was associated with a trend to a higher impact of plaque on PD. However, the WC contribution to PD, significant at low CRP, was no longer relevant at high CRP levels.

**Table 5:** Median regression on 50th percentile of PD as the dependent variable with plaque stratified by CRP risk categories, *ß* coefficients with 95% confidence intervals

Independent variables*	CRP < 1 mg/L *N* = 1370	*p*	CRP 1–3 mg/L *N* = 1228	*p*	CRP > 3 mg/L *N* = 768	*p*
Age, 10-year increments	0.03 (0.01; 0.06)	0.022	0.07 (0.03; 0.10)	< 0.001	0.06 (0.01; 0.10)	0.015
Female sex (ref. male)	0.02 (− 0.10; 0.13)	0.79	− 0.06 (− 0.19; 0.07)	0.35	− 0.05 (− 0.23; 0.13)	0.57
Plaque, 1st tertile	0	Ref.	0	Ref.	0	Ref.
Plaque, 2nd tertile	0.06 (− 0.00; 0.13)	0.067	0.05 (− 0.03; 0.13)	0.21	0.12 (0.00; 0.24)	0.044
Plaque, 3rd tertile	0.31 (0.23; 0.39)	< 0.001	0.38 (0.29; 0.46)	< 0.001	0.43 (0.30; 0.55)	< 0.001
HbA1c, %	0.04 (− 0.01; 0.09)	0.11	0.03 (− 0.02; 0.08)	0.18	− 0.02 (− 0.07; 0.04)	0.57
WC, 10-cm increments	0.09 (0.03; 0.16)	0.003	0.03 (− 0.03; 0.09)	0.31	0.01 (− 0.07; 0.09)	0.84

*Also adjusted for BMI, number of teeth, grip strength, smoking, education

## Discussion

Obesity and associated complications, especially in view of the increased prevalence of obesity-associated diseases and periodontal diseases, are of important public health concern. The prevalence of health implications of obesity and periodontitis will increase with rising life expectancy. In this population-based study, we confirmed that both obesity and periodontitis are associated with higher systemic CRP and fibrinogen levels with distinct differences regarding their relative contributions. Oral inflammation contributes differently to systemic CRP levels between different states of adiposity depending on whether obesity is present or not [7]. In this population, besides PD also plaque revealed a strong association with CRP along with the impact of obesity. In accordance with our results, only plaque and PD remained significantly associated with BMI after adjusting for different covariates but not with CAL when adjusted for age [20].

This finding may reflect the close relationship between factors favoring oral microbiota and obesity likewise. Such an association between obesity and oral bacterial-establishing subgingival biofilm indicates a possible link between oral microbiota and obesity [21]. Obesity is associated with increased levels of periopathogenic microbes residing in the biofilm constituting dental plaque [22]. Differences in the relationship between dental plaque and PD by adiposity states (Table 5) support such a hypothesis. In animal models, it was shown that periopathogenic microbes influence the gut microbiome influencing metabolic profiles probably related to obesity [23]. Moreover, increased inflammatory cytokines within the gingival crevicular fluid as measured in obese subjects were reduced by weight control [24]. Thus, there is evidence that oral microbiome has an influence on systemic characteristics, and, inversely, obesity affects inflammatory conditions in the oral cavity. PD is then eventually the periodontal outcome of a misguided inflammatory response contributing to systemic consequences, which may be superimposed by obesity-related inflammation.

A rich literature on the topic is available [25]. A recent experimental study has shown the comorbidity effect of obesity and periodontitis by demonstrating the elevation of the biomarkers associated with systemic inflammation, metabolic dysregulation, and periodontal destruction, when both disease processes coexisted, compared with each one independently [26]. In patients with periodontitis, inflammatory biomarker levels such as serum CRP are significantly higher than in healthy persons, while periodontal treatment reduced these levels [27]. Excess adiposity is one of the determinants of CRP elevation and together with periodontitis jointly associated with higher CRP levels in otherwise healthy adults [15, 28].

CRP is a nonspecific marker of inflammation produced predominantly by the liver in response to interleukin-6. Modest elevation of CRP can occur chronically, regarded as low-grade systemic inflammation, which has been associated with increased risks of cardiovascular disease, diabetes, or other adverse health outcomes, all conditions characterized by abdominal fat [29]. Likewise, periodontitis may also be involved in comorbidities of insulin resistance, type 2 diabetes mellitus, and cardiovascular disease commonly present in obese individuals. An imbalance towards a pro-inflammatory immune response could underline a bidirectional link between chronic periodontitis and other chronic diseases [30]. It is well established that periodontitis is associated with cardiovascular diseases and metabolic syndrome differently between men and women. Fibrinogen and white blood count similarly indicate that systemic low-grade inflammation might indeed represent one possible pathway for the effects of obesity, diabetes, or other chronic inflammatory conditions on periodontitis [31].

Insulin resistance was proposed to mediate the relationship between obesity and periodontitis, and hence, diabetes is to be taken into consideration [4, 8]. In a nationally representative U.S. sample of diabetes-free adults, periodontitis was associated with insulin resistance. Data on white blood cell count but not on CRP supported the role of inflammation as both mediator and effect modifier of the association [32]. In the present study, the contributions of HbA1c blood concentrations on CRP levels were attenuated when adjusted to waist circumference. However, the association between HbA1c and CRP was strengthened across the CRP distribution (Table 2). Moreover, muscle strength counteracts obesity-driven CRP increase (Fig. 1). Interaction between frailty, obesity, and muscle strength is important in an aging population and is also related to periodontitis and tooth loss [33, 34].

The oral inflammation contributes differently to systemic CRP or fibrinogen levels depending on whether adiposity is present or not. Inflammatory stimuli originating from obesity may override local contributions. A possible explanation for obesity overwhelming the effect of periodontitis can be seen in common pro-inflammatory pathways because adipose tissue participates actively in regulating systemic inflammation through the production of inflammatory adipokines and cytokines.

The sex-specific android or gynoid fat distributions are differently related to increased risks of systemic diseases such as coronary artery disease and diabetes, both of them also associated with periodontitis. Recently, it has been shown in a large population survey that even in normal weight individuals (BMI < 25 kg/m^2^) an android body shape with high WC is associated with an increased tooth loss [35]. Attitudes towards and biology of oral health are very different between men and women, resulting in sexual dimorphism in susceptibility to periodontitis. This is also true for the obesity periodontitis conundrum [36]. Visceral fat depots are consistently associated with increased concentrations of inflammation markers such as IL-6 and CRP, even after controlling for general adiposity [37]. Higher systemic CRP concentrations indicated increased risks of cardiovascular morbidity, diabetes, and metabolic syndrome more pronounced in women than in men [38, 39].

Our study has several limitations. There are the limitations inherent to cross-sectional study designs preventing data from drawing causal inferences about the relationship between obesity and CRP levels. WHR and waist circumference are surrogate markers for adiposity, and CRP is only a short living measure of inflammation induced by obesity as well as by periodontal inflammation, whereas fibrinogen has a longer half-life. Common confounding factors of obesity and periodontitis such as diet, health behavior, physical activity, and socioeconomic factors may be important [25]. It is also difficult to distinguish between chronic and acute origins of CRP, let alone different mechanisms characterized by markers other than CRP. Certainly, periodontitis and obesity are merely two ramifications within the more general principle of interactive and complex adaptions to internal and external challenges to which all organisms are subjected [40].

In conclusion, systemic inflammation may have a mediating function between adiposity and periodontitis. In any case, the efficacy of oral hygiene measures and the efficiency of weight reduction by diet and exercise may be good advice for keeping systemic inflammation low in order to prevent disease sequelae accompanying aging.

## Electronic supplementary material

ESM 1 (DOCX 41 kb)

## References

[1] Jimenez M, Hu FB, Marino M, Li Y, Joshipura KJ (2012). Prospective associations between measures of adiposity and periodontal disease. Obesity.

[2] Bastien M, Poirier P, Lemieux I, Després JP (2014). Overview of epidemiology and contribution of obesity to cardiovascular disease. Prog Cardiovasc Dis.

[3] Suvan J, D’Aiuto F, Moles DR, Petrie A, Donos N (2011). Association between overweight/obesity and periodontitis in adults. A systematic review. Obes Rev.

[4] Aoyama N, Suzuki JI, Kobayas hi N, Hanatani T, Ashigaki N, Yoshida A, Shiheido Y, Sato H, Kumagai H (2018). Japanese cardiovascular disease patients with diabetes mellitus suffer increased tooth loss in comparison to those without diabetes mellitus -a cross-sectional study. Intern Med.

[5] Christen T, Trompet S, Rensen PCN, Willems van Dijk K, Lamb HJ, Jukema JW, Rosendaal FR, le Cessie S, de Mutsert R (2019). The role of inflammation in the association between overall and visceral and subclinical atherosclerosis. Nutr Metab Cardiovasc Dis.

[6] Graßmann S, Wirsching J, Eichelmann F, Aleksandrova K (2017). Association between peripheral adipokines and inflammation markers: a systematic review and meta-analysis. Obesity.

[7] Torrungruang K, Katudat D, Mahanonda R, Sritara P, Udomsak A (2019). Periodontitis is associated with elevated serum levels of cardiac biomarkers – soluble ST2 and C-reactive protein. J Clin Periodontol.

[8] Chaffee BW, Weston SJ (2010). Association between chronic periodontal disease and obesity: a systematic review and meta-analysis. J Periodontol.

[9] Akram Z, Abduljabbar T, Abu Hassan MI, Javed F, Vohra F (2016). Cytokine profile in chronic periodontitis patients with and without obesity. A systematic review and meta-analysis. Dis Markers.

[10] Bastard JP, Maachi M, Lagathu C, Kim MJ, Caron M, Vidal H, Capeau J, Feve B (2006). Recent advances in the relationship between obesity, inflammation, and insulin resistance. Eur Cytokine Netw.

[11] Ebersole JL, Graves CL, Gonzalez OA, Dawson D, Morford LA, Huja PE, Hartsfield JK, Huja SS, Pandruvada S, Wallet SM (2016). Aging, inflammation, immunity and periodontal disease. Periodontology 2000.

[12] Tam BT, Morais JA, Santosa S (2010). Obesity and ageing: two sides of the same coin. Obes Rev.

[13] Marques-Vidal P, Bochud M, Bastardot F, Lüscher T, Ferrero F, Gaspoz JM, Paccaud F, Urwyler A, van Känel R (2012). Association between inflammatory and obesity markers in a Swiss population-based sample (CoLaus Study). Obes Facts.

[14] Cartier A, Coté M, Lemieux I, Perusse L, Tremblay A, Bouchard C, Després JP (2009). Sex differences in inflammatory markers: what is the contribution of visceral adiposity?. Am J Clin Nutr.

[15] Meisel P, Wilke P, Biffar R, Holtfreter B, Wallaschofski H, Kocher T (2012). Total tooth loss and systemic correlates of inflammation: role of obesity. Obesity.

[16] Völzke H, Alte D, Schmidt CO, Radke D, Lorbeer R, Friedrich N, Aumann N, Lau K, Piontek M, Born G (2011). Cohort profile: the Study of Health in Pomerania. Int J Epidemiol.

[17] Koliaki C, Liatis S, Dalamaga M, Kokkinos A (2019). Sarcopenic obesity: epidemiological evidence, pathophysiology, and therapeutic perspectives. Curr Obes Rep.

[18] Hong HG, Christiani DC, Li Y (2019). Quantile regression for survival data in modern cancer research: expanding statistical tools for precision medicine. Precis Clin Med.

[19] Bi X, Loo YT, Ponnalagu S, Henry CJ (2019). Obesity is an independent determinant of elevated C-reactive protein in healthy women but not men. Biomarkers.

[20] Benguigui C, Bongard V, Ruidavets JB, Sixou M, Chamontin B, Ferrières AJ (2012). Evaluation of oral health related to body mass index. Oral Dis.

[21] Zeigler CC, Persson GR, Wondimu B, Marcus C, Sobko T, Modéer T (2012). Microbiota in the oral subgingival biofilm is associated with obesity in adolescence. Obesity.

[22] Maciel SS, Feres M, Gonçalves TE, Zimmermann GS, Pereira da Silva HD, Figueiredo LC, Duarte PM (2016). Does obesity influence the subgingival microbiota composition inperiodontal health and disease?. J Clin Periodontol.

[23] Kato T, Yamazaki K, Nakajima M, Date Y, Kikuchi J, Hase K (2018). Oral administration of Porphyromonas gingivalis alters the gut microbiome and serum metabolome. mSphere.

[24] Park HS, Nam HS, Seo HS, Hwang SJ (2015). Change of periodontal inflammatory indicators through a 4-week weight control intervention including caloric restriction and exercise training in young Koreans: a pilot study. BMC Oral Health.

[25] Suvan JE, Finer N, D’Aiuto F (2018). Periodontal complications with obesity. Periodontology 2000.

[26] Virto L, Cano P, Jimenez-Ortega V, Fernández-Mateos P, González J, Esquifino AI, Sanz M (2018). Obesity and periodontitis: an experimental study to evaluate periodontal and systemic effects of comorbidity. J Periodontol.

[27] de Souza AB, Okawa RT, Silva CO, Araújo MG (2017). Short-term changes on C-reactive protein (CRP) levels after non-surgical periodontal treatment in systemically healthy individuals. Clin Oral Investig.

[28] Slade GD, Ghezzi EM, Heiss G, Beck JD, Riche E, Offenbacher S (2003). Relationship between periodontal disease and C-reactive protein among adults in the atherosclerosis risk in communities study. Arch Intern Med.

[29] Maiorino MI, Bellastella G, Giugliano D, Esposito K (2018). From inflammation to sexual dysfunctions: a journey through diabetes, obesity, and metabolic syndrome. J Endocrinol Investig.

[30] Sasaki H, Hirai K, Martins CM, Furusho H, Battaglino R, Hashimoto K (2016). Interrelationship between periapical lesion and systemic metabolic disorders. Curr Pharm Des.

[31] Pink C, Kocher T, Meisel P, Dörr M, Markus MRP, Jablonowski L (2015). Longitudinal effects of systemic inflammation markers on periodontitis. J Clin Periodontol.

[32] Demmer RT, Squillaro A, Papapanou PN, Rosenbaum M, Friedewald WT, Jacobs DR, Desvarieux M (2012). Periodontal infection, systemic inflammation, and insulin resistance. Diabetes Care.

[33] Leite MA, Morgenstern de Mattia T, Kakihata CMM, Bortolini BM, de Carli Rodrigues PH, Bertolini GRF, Brancalhao RMC, de Fatima Chasko Ribeiro L, Nassar CA, Nassar PO (2017). Experimental periodontitis in the potentialization of the effects of immobilism in the skeletal striated muscle. Inflammation.

[34] Eremenko M, Pink C, Biffar R, Schmidt CO, Ittermann T, Kocher T, Meisel P (2016). Cross-sectional association between physical strength, obesity, periodontitis and number of teeth in a general population. J Clin Periodontol.

[35] Kang J, Smith S, Pavitt S, Wu J (2019). Association between central obesity and tooth loss in the non-obese people: results from the continuous National Health and Nutrition Examination Survey (NHANES) 1999-2012. J Clin Periodontol.

[36] Meisel P, Eremenko M, Holtfreter B, Völzke H, Kocher T (2019). The sex paradox in the interplay between periodontitis, obesity, and serum C-reactive protein: data from a general population. J Periodontol.

[37] Beasley LE, Koster A, Newman AB, Javaid MK, Ferrucci L, Kritchevsky SB, Kuller LH, Pahor M, Schaap LA, Visser M (2009). Inflammation and race and gender differences in computerized tomography-measured adipose depots. Obesity.

[38] Lai MM, Li CI, Kardia SL, Liu CS, Lin WY, Lee YD, Chang PC, Lin CC, Li TC (2010). Sex differences in the association of metabolic syndrome with high sensitivity C-reactive protein in a Taiwanese population. BMC Public Health.

[39] Hu G, Jousilahti P, Tuomilehto J, Antikainen R, Sundvall J, Salomaa V (2009). Association of serum C-reactive protein level with sex-specific type 2 diabetes risk: a prospective Finnish study. J Clin Endocrinol Metab.

[40] Bennett JM, Reeves G, Billman GE, Sturmberg JP (2018) Inflammation – nature’s way to efficiently respond to all types of challenges: implications for understanding and managing “the epidemic” of chronic diseases. Front Med 5(613). 10.3389/fmed.2018.00316 10.3389/fmed.2018.00316 PMC6277637 30538987

