# Correction to: Competing interplay between systemic and periodontal inflammation: obesity overrides the impact of oral periphery

**DOI:** 10.1007/s00784-021-04033-0

**Published:** 2021-06-23

**Authors:** Peter Meisel, Christiane Pink, Vinay Pitchika, Matthias Nauck, Henry Völzke, Thomas Kocher

**Affiliations:** 1 Dental Clinics, Department of Periodontology, School of Dentistry, University Medicine Greifswald, Fleischmann-Strasse 42, D-17475 Greifswald, Germany; 2 Institute of Clinical Chemistry and Laboratory Diagnostics, Greifswald, Germany; 3 DZHK (German Centre for Cardiovascular Research), Partner Site Greifswald, University Medicine, Greifswald, Germany; 4 Institute for Community Medicine, University Medicine Greifswald, Greifswald, Germany

**Correction to** **:** **Clinical Oral Investigations**

**https://doi.org/10.1007/s00784-020-03514-y**

The article “Competing interplay between systemic and periodontal inflammation: obesity overrides the impact of oral periphery”, written by Peter Meisel, Christiane Pink, Vinay Pitchika, Matthias Nauck, Henry Völzke and Thomas Koche, was originally published Online First without Open Access. After publication in volume 24, issue 5, page 2045–2053 the author decided to opt for Open Choice and to make the article an Open Access publication. Therefore, the copyright of the article has been changed to © The Author(s) 2021 and the article is forthwith distributed under the terms of the Creative Commons Attribution 4.0 International License, which permits use, sharing, adaptation, distribution and reproduction in any medium or format, as long as you give appropriate credit to the original author(s) and the source, provide a link to the Creative Commons license, and indicate if changes were made. The images or other third party material in this article are included in the article’s Creative Commons license, unless indicated otherwise in a credit line to the material. If material is not included in the article’s Creative Commons license and your intended use is not permitted by statutory regulation or exceeds the permitted use, you will need to obtain permission directly from the copyright holder. To view a copy of this license, visit http://creativecommons.org/licenses/by/4.0.

The original article has been corrected.

